# Expressing one’s feelings and listening to others increases emotional intelligence: a pilot study of Asian medical students

**DOI:** 10.1186/1472-6920-13-82

**Published:** 2013-06-07

**Authors:** Keiko Abe, Phillip Evans, Elizabeth J Austin, Yasuyuki Suzuki, Kazuhiko Fujisaki, Masayuki Niwa, Muneyoshi Aomatsu

**Affiliations:** 1 Medical Education Development Centre, Gifu University School of Medicine, Gifu, Japan; 2 Department of Education for Community-Oriented Medicine, Nagoya University Graduate School of Medicine, Nagoya, Japan; 3 School of Medicine, College of Medical, Veterinary and Life Sciences, University of Glasgow, Glasgow, UK; 4 Psychology, University of Edinburgh, Edinburgh, UK

**Keywords:** Emotional Intelligence (EI), Personality trait, Asian medical students, Nationality, Gender

## Abstract

**Background:**

There has been considerable interest in Emotional Intelligence (EI) in undergraduate medical education, with respect to student selection and admissions, health and well-being and academic performance. EI is a significant component of the physician-patient relationship. The emotional well-being of the physician is, therefore, a significant component in patient care. The aim is to examine the measurement of TEIQue-SF in Asian medical students and to explore how the practice of listening to the feelings of others and expressing one’s own feelings influences an individual’s EI, set in the context of the emotional well-being of a medical practitioner.

**Methods:**

A group of 183 international undergraduate medical students attended a half-day workshop (WS) about mental-health and well-being. They completed a self-reported measure of EI on three occasions, pre- and post-workshop, and a 1-year follow-up.

**Result:**

The reliability of TEIQue-SF was high and the reliabilities of its four factors were acceptable. There were strong correlations between the TEIQue-SF and personality traits. A paired t-test indicated significant positive changes after the WS for all students (n=181, p= .014), male students (n=78, p= .015) and non-Japanese students (n=112, p= .007), but a repeated measures analysis showed that one year post-workshop there were significant positive changes for all students (n=55, p= .034), female students (n=31, p= .007), especially Japanese female students (n=13, p= .023). Moreover, 80% of the students reported that they were more attentive listeners, and 60% agreed that they were more confident in dealing with emotional issues, both within themselves and in others, as a result of the workshop.

**Conclusion:**

This study found the measurement of TEIQue-SF is appropriate and reliable to use for Asian medical students. The mental health workshop was helpful to develop medical students’ EI but showed different results for gender and nationality. The immediate impact on the emotional awareness of individuals was particularly significant for male students and the non-Japanese group. The impact over the long term was notable for the significant increase in EI for females and Japanese. Japanese female students were more conscious about emotionality. Emotion-driven communication exercises might strongly influence the development of students’ EI over a year.

## Background

Emotional intelligence (EI) is refers to an individual’s awareness on his or her own emotions, together with an awareness of the emotions in others and the ability to manage them and act appropriately. The term is usually attributed to Payne [1] who explored EI with respect to fear, pain and desire, and it was discussed in terms of personal intelligences [2] at a similar time. A robust explanatory framework as defined as *“a set of skills hypothesized to contribute to the accurate appraisal and expression of emotion in oneself and in others, the effective regulation of emotion in self and others, and the use of feelings to motivate, plan, and achieve in one's life.”*[3]. The concept was then linked to IQ and superior performance at work [4-6].

Instruments for measuring EI include those that conceptualize EI as a trait [7], and as an ability [8,9]. Trait EI strongly correlates with the “Big Five” personality traits (Neuroticism, Agreeableness, Openness, Extraversion, and Conscientiousness) [10]. The *Trait Emotional Intelligence Questionnaire* (TEIQue) [11] is composed of four factors (well-being, self-control, emotionality and sociability). Petrides and Furnham [7,11] proposed that the primary basis for discriminating between trait EI and ability EI is the measurement approach and not theoretical domains. The trait EI is measured through self-reported questionnaires, whereas ability EI should be measured through maximum performance tests with correct and incorrect answers.

The construct of EI assumes that the emotions are a significant resource, and that knowledge of them in oneself and in others promotes a more desirable out come in terms of satisfaction, achievement and well-being Consequently, there has been considerable interest in EI with respect to a number of issues related to medical education and patient care, including patient satisfaction, student selection and admission to medical school, student mental health and well-being, teaching specific topics, and academic performance. Reports upon the relationship between the physician’s EI and patient satisfaction [12] suggest that patient satisfaction is increased, and similar studies on Empathy [13,14] endorse the view [15].

The EI of undergraduate medical students appears to be associated with better health and life satisfaction [16], and there is some evidence that EI may mediate stress [17].

There is evidence that individuals with high EI cope better with emotional responses to work-stressors, and that these individuals have higher job satisfaction and lower stress levels, by “Keeping things in proportion” [18].

EI is highly responsive to training [11]. BEME Guide No. 17 reviewed educational programs to increase EI and summarized that the programs are more effective when using SPs, delivering interventions over short space of time, and emphasizing the empathic communication [19]. Recently, Stoller et al. [20] recommended teaching EI as part of the medical training curriculum because EI is a key competency for physician leaders.

Training in Emotional skills [21] varies considerably in terms of time, frequency and duration, and with varying kinds of intervention and conclusions, and whilst there is no one common activity, the conclusions suggest a positive outcome. Therefore, the authors wanted to establish if trait emotional intelligence could be improved and sustained, using a suitable intervention. One of the authors (KA) studied “The Healer’s Art” [22] which was sufficiently well understood as to be considered a valid intervention in the USA. The course focuses upon the humanistic aspects of professionalism engaging the students in making reflective explanations of shared narratives of life-events.

The aim is to validate the measurement of TEIQue-SF in Asian medical students and to explore how the practice of listening to the feelings of others and expressing one’s own feelings influences an individual’s EI, set in the context of the emotional well-being of a medical practitioner.

## Methods

### Sampling period

The participants were sampled pre-and post intervention and one year following the intervention

### Participants

The participants were taking part in The 5^th^ International Federation of Medical Students’ Association, Asia Pacific Regional Meeting, about Mental Health and Well-being held in 2007 [23]. All 181 delegates agreed to participate. They comprised 78 males with a mean age of 20.7 years (SD=2.24, range 17 – 31) and 103 females with a mean age of 22.3 years (SD=2.75, range 19–32). All participants were of Asian nationality: 69 Japanese, 41 Taiwanese, 41 Thais, and 30 Indonesian.

The time spent in medical school ranged between 1 and 6 years, with a mean of 2.3 years (SD=1.11, range 1–6), while for the group one-year post-survey, the mean was 3.7 years (SD=1.19, range 2–6). All participants agreed to participate and each individual was anonymised by allocation of a randomized number. In terms of the follow up online survey, 158 students received emails to participate in the survey. A summary of the characteristics of the follow-up sample is shown in the Table 1.

**Table 1:** Participants’ characteristics

		**Pre and post WS**	**Follow-up**
Gender	Male / Female	78 / 103	24 / 31
Age	Mean / range	20.7 / 17–31 (SD=2.24)	22.3 / 19–32 (SD=2.75)
Medical year	Mean / range	2.3 / 1–6 (SD=1.11)	3.7 / 2–6 (SD=1.19)
Nationality	Japanese	69	23
	Taiwanese	41	7
	Thais	41	13
	Indonesian	30	12

### Measures

The instruments were the 30-item TEIQue-SF (Trait Emotional Intelligence questionnaire-Short Form) [7,11] and a 20 items PT (Personality Trait) scale. The TEIQue-SF is a self-report questionnaire adapted from the full form of the TEIQue [11], which contains 15 facets. Two items from each facet were selected for the short version (TEIQue-SF). A Likert scale ranging from 1 (completely disagree) to 7 (completely agree) is used for the responses to the test items. The TEIQue is widely used and validated cross-culturally. Cooper and Petrides [24] indicated that the 4 higher-level factors of the TEIQue (Well-Being, Self-Control, Emotionality, Sociability) can be assessed using the TEIQue-SF, although they tend to have lower reliabilities than those derived from the longer scale. The TEIQue-SF does not however yield scores on the 15 trait EI facets, which can be obtained from the longer scale. The TEIQue-SF is suitable for the rapid assessment of global trait EI. The personality scale was a shortened form of the Saucier Minimarkers scale [25]. The scale contains 20 (reduced from 40 in the original scale to provide rapid assessment of personality) trait-descriptive adjectives, comprising 4 items for each of five dimensions of personality: Neuroticism (N), Extraversion (E), Openness (O), Agreeableness (A), and Conscientiousness (C). For each adjective, participants rate the extent to which the adjective describes them. A paper-based version of TEIQue-SF and PT was administered pre and post the workshop and a web-based version was administered one year after the workshop. The participants were also asked four questions to assess an effect of the workshop.

For statistic analysis, IBM SPSS 20.0 JP statistics was used. Gender and nationality, Japanese and non-Japanese (Indonesian, Taiwanese, and Thais) differences in the short term, pre and post workshop, were examined using ANOVA. A second analysis involved repeat-measures of analysis using data from all three time points with gender and nationality as between-subjects factors.

### Intervention

The workshop programme about mental health and well-being, as shown in Table 2, was delivered as a half-day workshop. The intervention was based on the work of Rachel Rehman [22]. The format begins with a short lecture about mental health and impaired healers. Two physician facilitators disclosed their own painful experiences, such as a junior resident’s sudden death. With these stories as an introduction, students worked in groups of 6 to disclose an experience in their own lives that was distressing, but not deeply personal. Self-introduction game followed by pairs of students working on issues of grief and loss. Emphasis was placed upon the importance of listening to and expressing feelings. Consideration was given to the emotional aspects of the experience under discussion, as a way of focusing upon the individual’s emotional intelligence.

**Table 2:** The programme of the mental health workshop

**Time**	**Contents**
13:00	Orientation
13:05	Self-introduction game: working in pairs
13:30	Grief and Loss: pair-work (Listening and Expressing feelings)
	1) What did your friends or parents do that was helpful?
	You have lost your most important possession. Something that you kept for years as a treasure. (ex: a picture or a present that you were given from your best friend. It is unique, the only one, and cannot be replaced) You looked for it for hours but in vain.
	You are at a loss what to do.
	2) What did your friends or parents do that was not helpful?
	On the contrary, what did they do that hurt you?
13:50	Sharing what you found
14:00	Mental health, What is grief?
14:20	Break
14:30	Impaired healers overview
14:50	Sharing 2 physicians stories: group work (Listening and Expressing feelings)
	Experience of sharing your own impaired story in your group
16:15	Closing

## Results

One hundred and eighty three international students participated in the workshop. One hundred and eighty one (99%) completed the paper-based survey at pre and post workshop. Invitations by email, to join the on-line survey, were sent to the 181 participants at the one-year follow up. Twenty-three emails were returned as ‘error’. Of the 158 participants who received an e-mail, 55 (34.8%) completed the online survey. As shown in Table 3, internal reliability (Cronbach’s alpha) was high for total EI (α= .89). Those for the 4 factors were acceptable. Correlations between the TEQue-SF, 4 Factors and the 5 personality traits are given in Table 4. Pearson’s correlations resulted moderate and weak correlations between TEIQue-SF and Personal trait except Openness. Two set of analyses were performed relating to 1) pre and post workshop (n=181) and 2) pre and post workshop and a 1-year follow up (n=55). Each set of samples was shown to follow a normal distribution by using a normality test (Kolmogorov-Smirnov test). Gender and nationality, Japanese and non-Japanese (Indonesian, Taiwanese, and Thais) differences in the short term, pre and post workshop, were examined using ANOVA. A second analysis involved repeat-measures of analysis using data from all three time points with gender and nationality as between-subjects factors.

**Table 3:** Reliability (Cronbach’s alpha) of Global trait EI and 4 factors of the TEIQue-SF

**Variables (items)**	**Pre-WS**			**Post-WS**		
	**Mean (SD)**	**α**	***F***	**Mean (SD)**	**α**	***F***
Global trait EI (30)	4.74 (1.85)	.871	16.80^**^	4.80 (1.77)	.891	13.76^**^
Well-being (6)	5.07 (.27)	.689	31.95^**^	4.58 (.12)	.456	146.51^**^
Self-control (6)	4.45 (.17)	.543	10.79^**^	4.51 (.24)	.661	9.34^**^
Emotionality (8)	4.76 (.19)	.655	8.81^**^	4.81 (.20)	.670	5.80^**^
Sociability (6)	4.52 (.23)	.651	4.19^**^	4.62 (.27)	.686	3.82^**^

^**^p <.01.

**Table 4:** Correlations between the TEIQue-SF and personality trait

**Personality trait (α)**	**Global trait EI**	**Well-being**	**Self-control**	**Emotionality**	**Sociability**
Neuroticism (.70)	-.37^**^	-.319^**^	-.431^**^	-.238^**^	-.182^*^
Extraversion (.49)	.227^**^	.218^**^	-.020	.189^*^	.288^**^
Openness (.54)	.129	.176^*^	.072	.100	.015
Agreeableness (.46)	.485^**^	.382^**^	.352^**^	.443^**^	.325^*^
Conscientiousness (.62)	.462^**^	.335^**^	.434^**^	.332^**^	.340^**^

^*^p <.05, ^**^p <.01.

### Effect size

A pre-test and post-test design with 1 year- follow –up was used to determine the effect of the intervention on participants EI. Small effect size was calculated by all medical students (Cohen’s d=.35).

### Comparisons of males and females and Japanese and non-Japanese groups between pre and post workshop

An EI score for each participant was calculated by summing up the item scores and the average item score, which is called Global Trait EI was also calculated by dividing this by the total number of items. The results at pre and post-workshop are shown in the Table 5.

1) Gender

With the data set 1, a paired t-test showed that EI scores for male students (p= .015) increased significantly but not for females post-workshop.

2) Nationality

**Figure 1:**
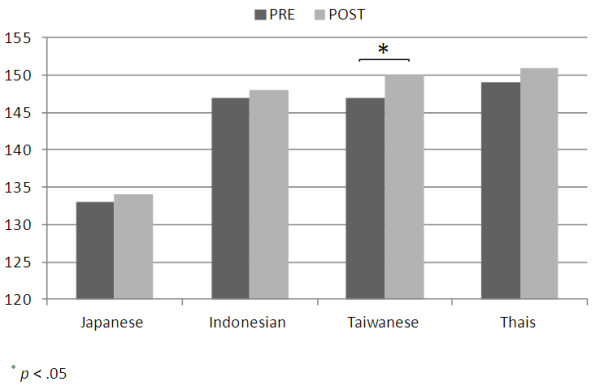
Comparison of EI scores of different nationalities at pre and post work shop (n=181).

**Figure 2:**
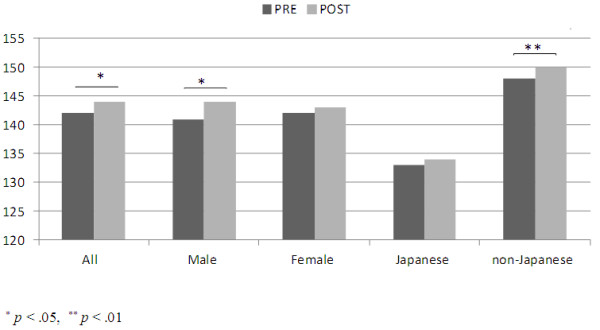
Comparison of males and females and Japanese and non-Japanese at pre and post work shop (n=181).

Dividing participants into Japanese and non-Japanese groups was examined initially, since it can be seen from Figure 1 that the three non-Japanese groups had very similar scores. Exploring the effect of nationality in more detail, 4 groups (Japanese, Indonesian, Taiwanese and Thais), as shown in Figure 1, were examined. The Japanese group scored much lower than other 3 nationality groups. An ANOVA analysis showed that these differences were significant (p< .001). As shown in Figure 2, there was a significant difference between Japanese and non-Japanese scores (p< .001) with Japanese scoring lower. The scores for non-Japanese increased significantly post-workshop (p= .007) but there was no significant difference between the pre- and post-workshop scores for Japanese.

**Table 5:** Average comparisons between pre- and post- workshop

**Group**	**Pre-WS M (SD),**	**Post-WS M (SD),**	**p -value**
	**Global Trait EI**	**Global Trait EI**	
Total (n=181)	141.92 (18.84), 4.73	143.69 (19.44), 4.79	.014
Male (n=78)	141.33 (20.13), 4.71	143.99 (19.43), 4.80	.015
Female (n=103)	142.38 (17.95), 4.75	143.46 (19.53), 4.78	*ns*
Japanese (n=69)	132.84 (16.15), 4.43	133.64 (17.27), 4.54	*ns*
Non-Japanese (n=112)	147.53 (18.26), 4.92	149.88 (18.12), 5.00	.007
Indonesian (n=30)	146.70 (18.78), 4.89	148.13 (18.29), 4.94	*ns*
Taiwanese (n=41)	147.02 (19.05), 4.90	149.71 (19.05), 4.99	.026
Thais (n=41)	148.63 (17.45), 4.95	151.32 (17.36), 5.04	*ns*

*ns* = not significant.

### Comparisons among pre, post workshop and one-year follow up

In the second data set, 55 students participated in all three surveys at pre, post WS and follow up. The mean scores for total EI and average of EI (Global trait EI) are shown in Table 6. Total EI scores were analyzed using repeated measures ANOVA followed by Bonferroni correction to account for multiple comparisons. Additionally to see if initial EI scores differed between the group followed up after one year (n=55) and those who were not followed up (n=118) scores were compared using an independent t test. There were no significant differences (p= .417).

1) Gender

**Figure 3:**
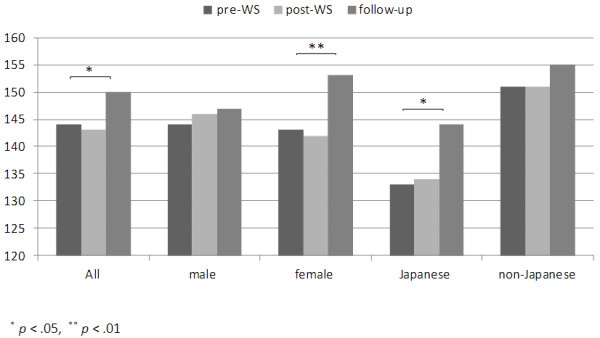
Comparison of males and females and Japanese and non-Japanese between pre and post workshop and follow-up.

Although the sample of the one-year follow-up group was reduced in size, there was a significant difference in their total EI (p= .034) over the 12 month period, as shown in Table 6. Their total EI scores increased by about 7 points at the follow up survey, as shown in Figure 3. The scores of female students increased significantly (p= .007). Effect size answered a medium size with all female students (Cohen’s d=0.55). In contrast, male students’ scores were significantly higher at post WS but did not show any further significant difference at the follow up period. The results of the Japanese medical students’ EI (n=23) on 4 factors showed, at the follow up period, there was a significant increase in Emotionality (p= .005). The EI for Japanese female students (n=13), showed a considerable increase (p= .023) over a year. Japanese female students’ effect size was calculated and showed a large effect size (Cohen’s d=1.21).

**Table 6:** Mean total scores of EI and average of EI at pre and post work shop and follow up

**Group**	**Pre-WS M (SD),**	**Post-WS M (SD),**	**A year follow-up M (SD),**	**p-value**
	**Global trait EI**	**Global trait EI**	**Global trait EI**	**(Repeated M)**
Total (n =55)	143.69 (18.82), 4.79	143 .42 (19.54), 4.78	150.45 (20.35), 5.02	0.034
Male (n=24)	144.33 (21.34), 4.81	145.71 (21.51), 4.86	146.83 (21.23), 4.89	*ns*
Female (n=31)	143.19 (16.97), 4.77	141.65 (18.04), 4.72	153.26 (19.52), 5.12	0.007
Japanese total (n=23)	133.35 (19.63), 4.45	133.48 (20.39), 4.45	144.22 (21.28), 4.81	0.031
Male (n=10)	131.90 (25.86), 4.39	133.8 (25.39), 4.46	130.9 (17.41), 4.36	*ns*
Female (n=13)	134.46 (14.19), 4.48	133.23 (16.68), 4.44	154.46 (18.49), 5.15	0.023
Non-Japanese (n=32)	151.13 (14.4), 5.04	150.56 (15.63), 5.02	154.94 (18.71), 5.16	*ns*
Male (n=14)	153.21 (14.79), 5.11	154.21 (13.6), 5.14	158.21 (15.91), 5.27	*ns*
Female (n=18)	149.5 (16.3), 4.98	147.72 (16.87), 4.92	152.39 (20.72), 5.08	*ns*

*ns* = not significant.

2) Nationality

Two groups, Japanese and non-Japanese, were analyzed using the one-year follow up data. The Japanese group showed a significantly increased EI score (p= .031) over a one-year period. Medium effect size was calculated by all Japanese medical students (Cohen’s d=0.53). In contrast, the non-Japanese showed a significant score difference between pre and post WS but scores for this group did not show a significant difference at the one-year follow up, suggesting a rapid shift followed by a period of stability.

3) Effects of the Mental Health workshop

**Figure 4:**
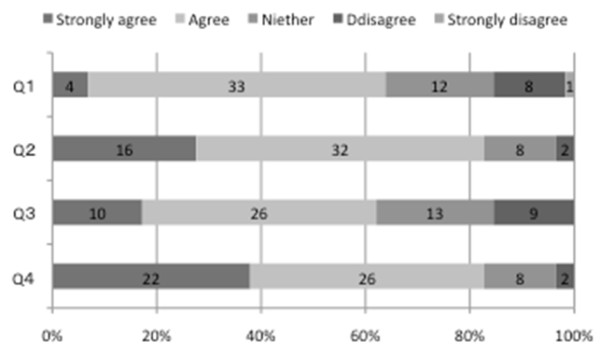
Effects of the mental health workshop (n=55).

With respect to the workshop, the participants were asked: “Please reflect on your experiences in the time following the workshop on the "Mental Health workshop" and indicate the extent to which you agree or disagree with each statement.” As shown in Figure 4, about 80% of students answered with either “strongly agree” or “agree” on Q2 and Q4. In terms of Q1 and Q3, about 60% of students answered either “strongly agree” or “agree” and about 16% answered with “disagree”.

## Discussion

This study is the first investigation of the TEIQue-SF among Asian medical students. It also investigated the influence of a half-day mental health workshop on the Emotional Intelligence of a group of international students, in the short and the long term, taking gender and nationality into consideration. There are 4 findings. Firstly, our analysis provided support for the reliability of the TEIQue-SF for Asian medical students. Although the reliabilities on 4 factors (range .661 - .689) of the TEIQue-SF were lower than for the full form, our results showed very similar values for the 4 factor scores compared to a previous study [11]. Also, high correlations of Global Trait EI and the 4 factors with the 5 personality traits were seen, with signs consistent with those found in other studies [7,11].

Secondly, Asian medical students’ EI increased following the intervention. As a short-term effect, immediately after the workshop, Global trait EI scores were significantly increased. Moreover, repeated measure ANOVA showed a significant improvement at 1-year follow up group (Global trait EI 5.02). Medium effect size showed that the mental health workshop made positive influences on EI of Asian medical students. This contrasts with Stratton et al [26], who reported that medical students EI decreased over time when comparing years 1 and 3.

Expressing one’s own feelings and listening to the feelings of others in the workshop required communication skills, emotional control and empathic ability. The result of this present study confirmed Austin’s previous study [27] that an exercise involving communication skills (talking with families) was significantly correlated with total EI.

Thirdly, ANOVA showed a significant difference in EI between Japanese and non-Japanese groups among Asian medical students. For Japanese medical students, Global trait EI (4.43) was significantly lower compared with participants from other Asian countries. The level of Global trait EI in Asian medical students (4.73) was similar to that found in UK university students, (4.72) [28]. But among UK students, the Global trait EI of students in natural sciences (medicine, chemistry/biology) was 4.76. Taking these results into consideration, it may be inferred that Japanese medical students’ EI scores (4.45) were especially lower than those of British medical students.

Japanese people have low self-efficacy [29,30] and tend to under-value their own ability. Trait EI is also named Trait emotional self-efficacy. Thus Japanese low self-efficacy may be reflected in the results of EI scores. In the present study however, Japanese Global trait EI matched the UK level in the 1-year follow up. A difference in the survey environment might be a contributing factor. Japanese people, especially females tend to be sensitive to interpersonal relationships [29] and tend to compare oneself with others so that they may lower their own self efficacy in the workshop where there were may expressive participants. In contrast, in the follow-up online survey, they answered the survey on their own private environment without other comparisons, which could be possible to estimate their self-efficacy.

It is acknowledged that the Japanese medical curriculum does not formally address issues of ‘death and dying’ or palliative care [31]. The previous study [32] reported that Japanese medical students’ empathy increased through medical school year in the cross sectional study. They decreased in the 4^th^ year and increased in the 6^th^ year. This may be accounted for by that the heavily science based curriculum in years 1 - 4 that has little patient contact until year 4. But medical students enjoy contact with patients in the clinical clerkship and this appears to contribute to the students’ growth in empathy. Thus, different curricula among countries may influence the EI development of medical students’.

Our findings support the assertion that talking with others about difficult problems improves an individuals’ EI. The participants had attended medical school for 2.7 years (mean) and, therefore, had had relatively little experience in meeting patients. Attending an international meeting and talking in English may help medical students open up their feelings, where talking about emotional driven issues in English may be a unique and impressive experience in itself. The impact of the workshop was sufficiently strong to compensate for lack of experience, and sufficiently strong to encourage EI development over a longer period. Thus, such communication exercises should be included in the early stages of medical education.

Fourthly, there is a gender-related time factor. Male students improved their EI immediately after the WS but did not improve further over the following year. In contrast, female students did not show much difference at the post workshop stage. However, their EI, especially EI in Japanese females, had improved significantly a year later with a large effect size. This extra time may be required to allow Japanese females to increase their emotional self-efficacy. It has been reported that female EI is higher than male EI in UK citizens where the Global trait EI sore was 5.18 for women and 5.02 for men [24]. Our results imply that the intervention influenced the students over the year following the workshop.

In terms of gender difference, male students showed a more positive result on EI than female students in the short term, however the female students, especially Japanese females showed greater improvement at the 1-year follow up. A previous study [27] reported that male medical students increased and female decreased in empathy between year 1 and 2, in a context of a conventional curriculum, with no intervention. This provides a point of reference that suggests that an intervention would be a beneficial exercise in the pre-clinical stage of the curriculum.

Emotionality and Well-being in 4 factors of the TEIQue-SF of Japanese female medical students showed significant differences and these factors are highly correlated with conscientiousness. These results imply that Japanese female students are more conscious about emotions and may need more time or more experience to develop their ability to deal with them, while male students seem to respond immediately. The reasons for this are not clear, and further investigation is required about this particular issue. However, Petrides [33] indicates that self-reporting has a gender bias, which support the assertion that female Japanese students require further experience to be confident about self-reporting a change in their feelings.

Answers to the questions indicated high levels of satisfaction with the workshop, with 80% admitting that they would recommend the workshop to a friend. Participants reported improved listening and expression skills, and using techniques for dealing with difficult situations more readily. These findings from the follow up questions related to the workshop also imply that participants are still conscious about these emotion-driven communication skills. This helps to explain why participants’ EI increased at the follow up survey. The overall implication is that the workshop was appropriate and helpful in developing medical students’ Emotional Intelligence.

### Limitations

Ideally the mental health workshop, should have been conducted in a small group of about 30 students. However, the method of sampling was restricted by the numbers attending the conference and the practicalities of distributing and returning the questionnaires to anonymous sub-groups in a conference setting. The sample bias is recognized. The students who attended the workshop were highly motivated and probably had very positive attitudes. The follow-up survey 1-year on, had a reduced response rate, in part because some of the participants’ email addresses were no longer used, and those who replied may have been more highly conscientious and compliant. There may exist bias in assessment between using the paper based and web-based survey. A larger study, that includes self-reporting linked with a third-party evidence, and a control group, is now required to test this assertion.

## Conclusions

The measurement of TEIQue-SF is appropriate and reliable to use for Asian medical students. The difference in the scores between the pre- and post-workshop suggests that an intervention can make an immediate impact on the emotional awareness of individuals, and this was particularly significant for male students and the also students who are not Japanese. The impact over the longer term is notable for the significant increase in EI scores for females and Japanese medical students who did not have very much experience of patient contact. This pilot study suggests that a mental health workshop focusing on expressing one feelings and listening to others is an appropriate and helpful intervention for students in the early years of medical training and can raise levels of awareness of emotional intelligence in an individual, and that this can be sustained over a period of at least one year.

### Ethical considerations

The subjects were international students attending the workshop. At the time of conducting this work, formal approval from an Ethics Committee is not normally required in this region. However, permission to conduct the research was granted by the International Medical Student Committee. A written explanation about the purpose of the research was given to each student, and they were invited to contribute, and given an option to withdraw without penalty, if they wished. As this was a written survey a completed form was accepted as consent. They were informed that the results would not contain any information that would allow individuals to be identified.

The data was gathered under the auspices of the International Federation of Medical Student’s Association, at Osaka, Japan. Data was analyzed and discussed in an iterative process at the Universities of Gifu, Nagoya, Glasgow and Edinburgh.

## Competing interests

The authors declare that they have no competing interests.

## Authors’ contributions

KA was the primary investigator. PE contributed to the planning, the distribution of the follow-up questionnaires and contributed to writing the paper. EA provided guidance and advice about the selection and application of the instruments, and interpretation of the results. YS and MN contributed data analysis and interpretation of the result. KF and MA contributed to the planning and conducting the workshop and distribution of the pre-post workshop questionnaires and also interpretation of the results. All authors read and approved the final manuscript.

## Pre-publication history

The pre-publication history for this paper can be accessed here:

http://www.biomedcentral.com/1472-6920/13/82/prepub
